# The macrodomain family: Rethinking an ancient domain from evolutionary perspectives

**DOI:** 10.1007/s11434-013-5674-9

**Published:** 2013-03-14

**Authors:** XiaoLei Li, ZhiQiang Wu, WeiDong Han

**Affiliations:** Department of Molecular Biology, Institute of Basic Medicine, School of Life Sciences, General Hospital of the Chinese People’s Liberation Army, Beijing, 100853 China

**Keywords:** macrodomain family, environmental stress, biological evolution, functional diversity

## Abstract

The reasons why certain domains evolve much slower than others is unclear. The notion that functionally more important genes evolve more slowly than less important genes is one of the few commonly believed principles of molecular evolution. The macro-domain (also known as the X domain) is an ancient, slowly evolving and highly conserved structural domain found in proteins throughout all of the kingdoms and was first discovered nearly two decades ago with the isolation and cloning of *macroH2A1*. Macrodomains, which are functionally promiscuous, have been studied intensively for the past decade due to their importance in the regulation of cellular responses to DNA damage, chromatin remodeling, transcription and tumorigenesis. Recent structural, phylogenetic and biological analyses, however, suggest the need for some reconsideration of the evolutionary advantage of concentrating such a plethora of diverse functions into the macrodomain and of how macrodomains could perform so many functions. In this article, we focus on macrodomains that are evolving slowly and broadly discuss the potential relationship between the biological evolution and functional diversity of macrodomains.

## 1 Macrodomains: A versatile, evolutionarily conserved family

The highly evolutionarily conserved macrodomains were first discovered nearly two decades ago with the isolation and cloning of *macroH2A1* [1], which has since been shown to have alternatively spliced forms [2]. MacroH2A is highly conserved in all vertebrates; furthermore, macrodomains can be found in all of the phylogenetic kingdoms, and these domains have evolved common and fundamental roles in the control of biological processes, indicating that many of these related proteins have existed since the beginning of evolution. To date, most of the members of the macrodomain family are conserved throughout evolution (Figure 1(a) and (b)), with homologs identified in viruses (*Coronaviruses* and *Alphaviruses*), archaea (*Archaeoglobus fulgidus*), bacteria (*Escherichia coli*), invertebrates (*Drosophila melanogaster*), amphibians (*Xenopus laevis*), mammals (*Homo sapiens* and *Mus musculus*), and plants (*Arabidopsis thaliana* and *Oryza sativa*) [3]. Indeed, as annotated by the SMART database, macrodomains have been identified in over 300 different proteins in all organisms, ranging from thermophiles to humans [4]. In humans, at least 10 genes encoding macrodomain-containing proteins are found, and each protein contains one to three macrodomains. Although macrodomain-containing proteins are products of different genes, they all contain the defining characteristics of the macrodomain [5]. The "macro" module is a roughly spherical protein domain of approximately 25 kD, which is composed of seven parallel and antiparallel β-sheets connected via six α-helices. Much of the research on the structure of the macrodomain has focused on the conservation of its active site (ligand-binding sites), as supported by the several

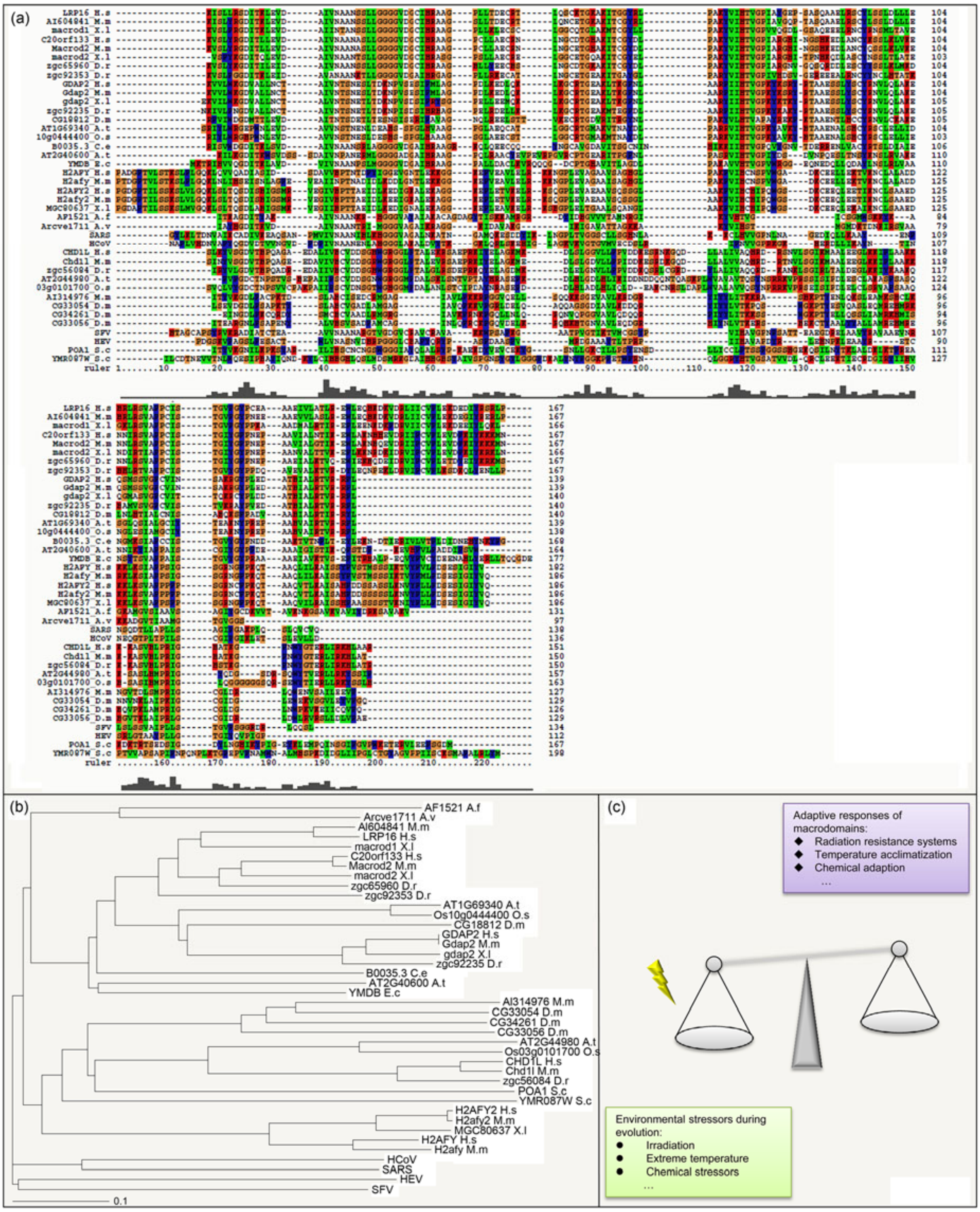

**Figure 1** Alignment and evolutionary tree of macrodomains in different organisms. (a) Multiple sequence alignment of selected macrodomains. An alignment of macrodomain orthologs from diverse species prepared using ClustalX. Protein identifier codes include the following abbreviations: mammalia (*Homo sapiens*=H.s., *Mus musculus*=M.m.); non-mammalian vertebrates (*Xenopus laevis*=X.l., *Danio rerio*=D.r.); invertebrates (*Drosophila melanogaster*=D.m., *Caenorhabditis elegans*=C.e.); plants (*Arabidopsis thaliana*=A.t.; *Oryza sativa*=O.s.); fungi (*Saccharomyces cerevisiae*=S.c.); bacteria (*Escherichia coli*=E.c.); archaea (*Archaeoglobus fulgidus*=A.f.); and viruses (SARS, SFV, HCoV, and HEV). All of the protein sequences that contain a macrodomain were extracted from the National Center for Biotechnology Information (NCBI), and the longest isoform for each gene was used. The sequence conservation is plotted beneath the alignment, and conserved residues are marked and color coded according to the default ClustalX settings. Amino acid numbers for the macrodomains are indicated. (b) A neighbor-joining tree is shown that is based on the protein sequence of the macrodomain. The relationships shown in the tree are based on the multiple sequence alignment using ClustalX as the alignment tool. The branch lengths are proportional to the mutation rate. (c) Schematic illustration of the balance between the selective pressure acting on organisms and the adaptive responses during the course of evolution. Environmental stressors could serve as the driving forces of protein evolution. During evolution, macrodomains appear to be important for maximizing cell survival upon the exposure to stresses and for cross-protection against unrelated stresses.

recently determined structures of the macro domains of human macroH2A1.1 and *Archaeoglobus fulgidus* Af1521 [5]. Recently, structural, enzymatic, and binding studies using new resources and technologies have indicated that macrodomains function as binding modules for the metabolites of $NAD^+$, including poly (ADP-ribose) (PAR), which is produced in reactions catalyzed by PAR polymerases [6]. However, although there is a high degree of sequence similarity within the different macrodomains, particularly for those residues that might be involved in substrate binding, not all of the macrodomain-containing proteins possess the capacity to bind PAR. The reason for this, at least in part, lies in the sequence variation among the macrodomains, which might be responsible for the functional specificity of the individual proteins.

Macrodomains are found as modules of multidomain proteins but can also constitute a protein alone. Although reports of the existence of cellular macrodomain-containing proteins only appeared in the literature two decades after the initial discovery of macroH2A, the distribution and evolution of macrodomains have been studied in diverse organisms in recent years. The studies to date indicate that the majority of eukaryotic macrodomain proteins are multimodular, comprising various regulatory and signaling modules. Indeed, extensive fusion and recruitment events of non-macrodomain proteins are observed in many members of the macrodomain family. These domain architectures play key roles in specificity and targeting and in establishing molecular connections and, hence, are vital to the understanding of the biological functions of macrodomain proteins. As documented for other protein families, the macrodomain family represents an excellent example of proteins with functional diversification that is achieved by versatile modular organization. In this minireview, we will discuss how macrodomains can perform many functions and what the evolutionary advantage is of concentrating such a plethora of diverse functions into this domain family.

## 2 How could macrodomains perform so many functions?

According to the general understanding, evolution is a process of the gradual change of a system, from a simpler to a more complex state. Macrodomains are one such system, and one of the challenges for modern protein domain science is to outline those earlier stages that, presumably, preceded the modern state. Proteins have existed for billions of years, and they have developed a plethora of structures and functions. Because "evolutionary pressure acts on the entire organism, rather than on a particular molecular" [7], from an evolutionary perspective, protein evolution cannot be viewed as an independent process. Accordingly, the understanding of the origin and possible interconversion of protein domains within the framework of Darwinian evolution has long appeared to researchers to be one of the most complex riddles in molecular bioscience. However, the conclusion of the human genome sequencing project has allowed a glimpse into the past by permitting the comparison of proteomes and their differences in terms of the protein content and modularity. Many groups have recently investigated protein evolution with a focus on precisely how new protein-coding genes may actually arise, and, furthermore, whether these genes are under strong selective pressure, thus indicating the high innovative potential of organisms. Although there is yet no consensus regarding how general and widespread the mechanisms are, it appears that the first step in the formation of a new protein-coding gene is often the emergence of an RNA gene, which may be followed by the creation of an ORF [8–10] and the subsequent introduction of introns [10].

Macrodomain proteins are functionally promiscuous and are implicated in the regulation of diverse biological functions, including DNA repair, chromatin remodeling and transcriptional regulation [3]; how macrodomain proteins could perform so many functions, though, remains unknown. From a domain evolution perspective, the most plausible explanation to this question lies in the fact that, by further concatenation, macrodomains can assemble into more complex multifold proteins with other subdomains. Accordingly, the domains can be viewed as the building blocks of proteins, and, with the exception of some disordered proteins, all proteins consist of one or more domains [11]. During evolution, different domains have been duplicated, fused and recombined to produce proteins with novel structures and functions [12]. Macrodomains have long been postulated to evolve by the rearrangements of larger fragments, which typically coincide with what is structurally defined as domains or structural motifs. Thus, the changes in domain architecture are underlined by significant alterations at the genetic level. Examples of the molecular mechanisms that can direct these rearrangements are gene fusion and fission [13], alternative gene splicing and retropositioning [14], and exon shuffling through intronic recombination [15]. However, even though there is evidence that changes in the protein domain composition are directed by gene fusion and fission in prokaryotes [16], the exact mechanisms that underlie these changes in eukaryotes remain unknown [13,14]. Apart from being dependent on the mechanisms that determine them, the existing domain combinations are also the result of selective forces that enable them to remain in a given population. Interestingly, some domains that are observed in a number of different domain combinations are considered to be promiscuous and are, typically, involved in protein-protein interactions; some of these domains also play important roles in signaling pathways. As noted recently, most of the macrodomain proteins also contain a plethora of diverse additional domains, allowing them to interact with specific target proteins or nucleic acid regions [3]. Given the large number of genes in the human genome and the comparatively small number of domains, the extensive combination, mixing and modulation of the existing domains has occurred during evolution to generate the multitude of functions necessary to sustain life. Notably, most of the members of the macrodomain family contain additional domains that mediate protein-protein (the WWE domain) or protein-lipid (the SEC14 domain) interactions or act as chromatin-remodeling enzymes (the SNF2 domain) [3]. In addition, the presence of a macrodomain in the histone protein, macroH2A, and in proteins containing DNA- and RNA-binding motifs would suggest an essential role in nucleic acid recognition. Therefore, we do not find a lone macrodomain but rather a diversity of macrodomain-containing proteins. Often, merely a few macrodomain proteins per cell are found to associate with specific protein partners, other transcriptional factors or chromatin regions. This fact, together with evidence that suggests that not all of the macrodomain proteins possess the same capabilities, implies that these macrodomains were able to become promiscuous in the first place because they had the potential to be useful within various contexts.

Domains are compact regions of protein structure that often confer distinct functions. The domain architecture, or the order of domains in a protein, is frequently considered as a fundamental level of the functional complexity of the protein [17]. Thus, it will be of interest to determine whether the different members of the macrodomain family might have a redundant function or completely distinct roles. For instance, knockout (KO) mice that lack macroH2A1 develop normally [18], whereas macroH2A-deficient zebrafish, which express only one form of macroH2A (macroH2A2), show developmental defects [19]. Therefore, in light of the two completely different results, there might be additional regulatory pathways that compensate for the loss of macroH2A function in some organisms (e.g., mice). One might speculate that the two forms of macroH2A would compensate for each other. Evidence is gradually emerging, and many details remain to be elucidated. For instance, there is no direct evidence that the macrodomain in macroH2A plays a major role in the regulation of development. Thus, in the future, animals with reduced macrodomain dosages can be used to test whether the different macrodomain modules could functionally compensate for each other. It is these types of animal model systems that will ultimately allow us to determine the precise role of macrodomains during development. MacroH2A is associated with transcriptional repression, but new evidence seems to indicate that the different macroH2A subtypes do not behave in the same way and, consequently, may perform different functions. In addition, the differential expression patterns of the macroH2A subtypes have been reported, which strongly supports the idea that the different subtypes operate in a diverse and functionally opposing manner [20]. More recently, the importance of macrodomains in mediating apoptosis has been validated by many groups, and accumulating data indicate an antagonizing role for the eukaryotic macrodomains in apoptosis [21–23]. One question that is invoked is why such a large amount of cellular energy should be spent on the production of many macrodomains with similar functions and whether different members of the macrodomain family have redundant functions. An interesting and possible answer to this query is that, during evolution, a role for macrodomains in the regulation of cell apoptosis occurs in response to biological, chemical or physical stimuli; therefore, upon exposure to different stimuli, organisms could depend on non-mutually exclusive mechanisms via the different macrodomains to inhibit apoptosis.

## 3 What drives macrodomain evolution?

Although distinct evolutionary pressures might operate in different organisms, a slow evolution of macrodomain proteins appears to be occurring in several diverse taxonomic groups. The fact that macrodomains are involved in many crucial biological processes poses interesting questions for evolutionary biologists: why are macrodomain genes evolving so slowly, and what is the functional consequence of this slow evolution? We propose that the selective forces of solar irradiation, climate and chemicals could both directly and indirectly and individually or in combination provide the evolutionary forces that drive the slow evolution of macrodomains (Figure 1(c)).

### 3.1 Counter-extreme environments

Evolutionary adaptation might be the only way that threatened species can persist if they are unable to disperse naturally, and adaptation is also a crucial theme in evolutionary biology: one of the most prominent features in the history of life is adaptive radiation. The early environment on Earth is a matter of conjecture; however, by piecing together evidence, it is currently believed that the early environment on Earth featured frequent high-energy events, such as volcanic eruptions, continuous torrents of rain, and abundant lightning. There was little, if any, oxygen in the atmosphere of ancient Earth and certainly no ozone layer, thus allowing ultraviolet radiation from the sun to reach the Earth's surface. Moreover, when the first forms of life appeared on Earth, there was no ozone layer to protect these organisms from ultraviolet exposure. Early adaptations included the development of pigments to protect against the ultraviolet radiation, then outer tissues to protect the internal tissues and the development of repair mechanisms, including DNA repair and genome maintenance. All living organisms, from bacteria to archaea to eukaryotes, have an impressive number of proteins and pathways that help to maintain the integrity of the genome and the high-fidelity of replication during growth [24]. Indeed, such repair systems persist today. When ultraviolet or other ionizing radiation damages DNA, repair enzymes remove the damaged portion and replace it with normal DNA. Tellingly, studies on macrodomains have indicated that, in some cases, these domains might contribute to DNA repair in mammalian cells after the exposure to DNA-damaging agents [22,25]. Specifically, the origin of macrodomains was examined by the comparison of the protein sequences in viral, bacterial, archaeal, and eukaryotic organisms, and the multiple sequence alignment indicates that there is a high level of sequence homology among these organisms. Such comparisons indicate that the macrodomain is derived from a gene that originated prior to the appearance of eubacteria and eukaryotes and suggest that this domain has retained the basic function of its ancestor. What is the evolutionary advantage of the macrodomain? One plausible explanation lies in the fact that the macrodomain might counteract and restrict DNA damage at multiple levels and in different ways: by mediating the rearrangement of chromatin and transiently affecting the DNA-damage response in a PAR-dependent manner; by actively regulating DNA repair; and/or by integrating DNA repair with checkpoint responses [3].

Adaptive changes are likely to influence the ability of species to take advantage of the potentially favorable conditions arising from extreme environments. However, with few exceptions, the importance of evolution tends to be ignored both in broader discussions about the effects of climate change on biodiversity and in models for predicting species' responses to climate change. Climate change has already led to alterations in the distribution of species, phenotypic variation, and allele frequencies [26,27]. Although such connections will provide an important insight, the physiological mechanisms underlying these trends remain uncertain. In fact, the precise physiological and biochemical mechanisms that define the thermal limits of species are often still unknown, in spite of our extensive understanding of how temperature affects the physiology and biochemistry of organisms. This uncertainty of the mechanism raises the question of how molecular adaptations could lead to physiological plasticity in response to a physical driver, such as temperature, and whether changes in the environmental temperature could drive the evolution of macrodomains. The answer may be that parallel or branched signaling pathways activate distinct suites of temperature-acclimation responses. Biologically, the adaption to cold or warm conditions is complex, involving dramatic changes in gene expression, and the results from functional genomic approaches have revealed the transcriptomic responses of organisms while they are experiencing variations in temperature. Accordingly, transcriptomic analyses may reveal the changes in gene regulatory networks that disclose the potential plasticity in response to changing environmental factors [28]. Notably, the expression of the histone macroH2A variant is drastically regulated during the temperature-acclimatization process. Using immunofluorescence assays, a stronger signal of the macroH2A protein is visible throughout the nuclei of cells during the winter compared to the summer, which demonstrates that macroH2A expression is drastically upregulated during cold acclimation [29]. Previous studies have indicated that macroH2A may accumulate in constitutive heterochromatin, could contribute to maintain its repressed state and could contribute to transcriptional silencing by acting in synergy with other repressive markers, such as DNA methylation and histone deacetylation and methylation [30]. Furthermore, the macrodomain of macroH2A may be involved in the ADP-ribosylation of chromatin, with potential implications for transcriptional silencing. During winter conditions, the histone macroH2A variant could be an important factor for the global reorganization of chromatin regions and the regulation of gene expression during the acclimatization process. Altogether these data provide an interesting early glimpse into how our environment can modulate the state of chromatin through altering the expression and incorporation of macrodomain histone variants. It is tempting to speculate that temperature, serving as a driver, could contribute to macrodomain evolution because rapid temperature change is likely to produce a range of selective pressures on populations. Increasing periods of temperature stress will produce a directional selection for resistance, particularly in species that exist in a state close to physiological limits such as during cold acclimatization. Being that macrodomains evolve slowly, it is, thus, possible that throughout evolution these domains have a major influence on the capacity of organisms to acclimatize to new environmental conditions.

### 3.2 Responses to chemical signals

Chemical signals, whether in the form of amino acids (and their derivatives), polypeptides, steroids, or nucleotides, are used to communicate information to cells at all stages of their life cycle. These signals inform the cells when it is time to change the rates of various activities, when to progress through developmental change, and in some cases, even when to die. To date, the common understanding of the evolution of organisms is that it is a process from simple, unicellular organisms to complex, multicellular life forms. Furthermore, similar means of communication are employed by plants, multicellular invertebrates, and single-celled organisms, such as protists and bacteria [31]; indeed, the use of chemical signals for communication between cells is a universal strategy. Specifically, signaling molecules, also referred to as autoinducers, which themselves carry no intrinsic message of a universal nature, bind to receptors on or in cells, leading to changes in gene expression at some threshold concentration to adapt to their environment. In principle, the spatial and temporal organization of the molecules within a cell is critical for coordinating the many distinct activities performed by the cell. Although most studies on macrodomains concentrate on their essential roles in different signaling pathways, macrodomains have been found to play central roles in physically assembling the relevant molecular components in an increasing number of biological processes. Because the transactivation potential of a transcription factor depends on the cofactors that it recruits, in the past two decades, a great deal has been learned about the functions mediated by macrodomain cofactors. For instance, macrodomains act as transcriptional cofactors in a series of signaling pathways regulated via various chemical signals, including IL-4 [32,33], TNF-$\alpha$ [23], and hormones [34–36]. Intriguingly, in addition to their function as cofactors of specific transcription factors, our group has identified a macrodomain protein, termed LRP16, which is regulated via a hormone-dependent manner, and we also established the existence of a feedforward mechanism between the macrodomain and hormone signals [35,36]. From evolutionary considerations, why do macrodomains play essential roles in the response to different chemical signals? It is possible to imagine that chemical defenses and cell-cell communication are important determinants of survival for organisms. After exposure to particular chemicals, a macrodomain could respond rapidly to resist these chemicals by regulating different signaling pathways. However, communication and defense will evolve and remain stable only if organisms gain benefits from this process [37]. Because rapid evolution implies a cost, nature will select against such unnecessary waste. Thus, it is easy to imagine that why macrodomains evolve much more slowly than other domains during the course of evolution.

## 4 Speculations

Two decades ago, we might have never imagined that the sequences of the macrodomains from unrelated species would be so similar and that their evolution would be directed by an adaptive change. Certainly, there is more interest today in the molecular evolution of proteins than at any time in the past. Although we might speculate that we have gained much knowledge regarding the slow evolution of many macrodomains, much more work needs to be conducted. The principle of evolution is to favor organisms that fit their environment through minimal costs; accordingly, organisms would manipulate many functions into a single class of proteins/domains over time. Therefore, it might be expected that, at large spatial scales and over millions of years, the functional diversity of macrodomains might reflect the complex interplays between abrupt and gradual environmental changes, the varying thresholds in dynamic equilibria, and the interactions between species (Figure 2).

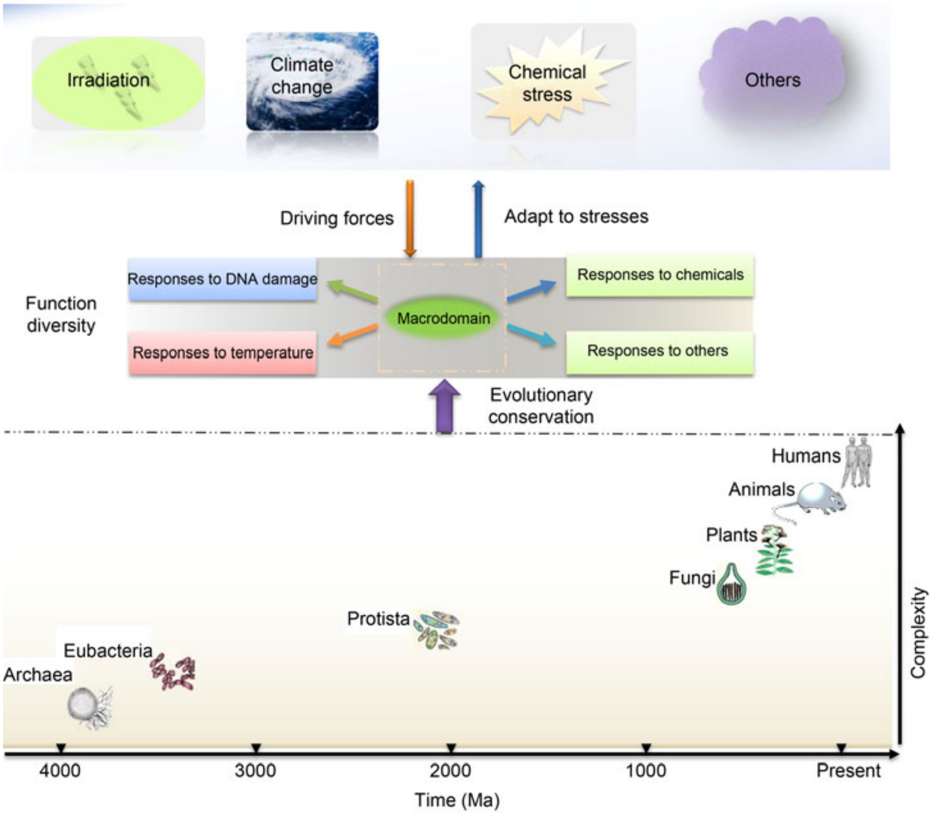

**Figure 2** Putative scenarios for the evolution of macrodomains. This graph is intended to present an overview and conjecture; some dates are still being debated, and the abscissa (“complexity”) has an arbitrary scale. The figure depicts the origin of the macrodomain and the driving forces of evolution. Schematic representations are shown for a small number of model organisms from each of the three domains of life. The macrodomains are highly evolutionarily conserved from unicellular to complex, multicellular organisms, and we speculate that many environmental stresses could have contributed to the evolution of macrodomains throughout the history of life on Earth. To adapt to stresses, life forms might concentrate such a plethora of diverse of functions into macrodomains.

The sequences of the macrodomains from a wide variety of species have been surveyed. Although we must consider that sequence differences in highly evolutionarily conserved macrodomains within the same population of the same species do exist, it is unclear how this is possible. One potential explanation is that, as described by neutral theory [38], mutations occur and even become fixed with no apparent effects. The existence of "highly evolutionarily conserved macrodomains" is surely related to the neutral theory [38]. On the other hand, explanation for the existence of sequence variation within the same population requires arguments based on population genetics. Mutation occurs in the gene encoding the domain in the same rate as in other genes, but most of them are ultimately removed from population and therefore the domain is highly conservative in the evolutionary time-scale. However, such mutants may be maintained for a while in the population if they are not very deleterious, the sequence variation of the macro domain we observed may represent such a case. Although the situation may be much different from the macro domain family, the difference between within-species comparison and between-species comparison observed by Hasegawa et al. [39] might be relevant to this problem. The study by Hasegawa et al. has indicated that in conserved genes, nonsynonymous rates within species tend to be higher than the between-species rates by a greater proportion than in fast-changing genes [39]. Thus, further experimental evolutionary studies on macrodomains should be performed to help clarify both the reasons why macrodomains show such extensive sequence conservation and the role of this conservation in the speciation process. We believe that the unique perspective of the temporospatial evolution of macrodomains presented in this review will foster the development of new ways to study other proteins that evolve slowly and the relationships between their structure and function.

*This work was supported by the National Natural Science Foundation of China (31201033, 31270820, 81071617 and 81230061) and in part by the Ministry of Science and Technology of China (2012CB518103 and 2012CB518105). We apologize to our many colleagues whose work could not be cited due to space constraints. We thank the members of our labs for critically reading the manuscript.*

1 Pehrson J R, Fried V A. MacroH2A, a core histone containing a large nonhistone region. Science, 1992, 257: 1398–1400
2 Kustatscher G, Hothorn M, Pugieux C, et al. Splicing regulates nad metabolite binding to histone MacroH2A. Nat Struct Mol Biol, 2005, 12: 624–625
3 Han W D, Li X L, Fu X B. The macro domain protein family: Structure, functions, and their potential therapeutic implications. Mutat Res, 2011, 727: 86–103
4 Letunic I, Copley R R, Schmidt S, et al. Smart 4.0: Towards genomic data integration. Nucleic Acids Res, 2004,32: D142–144
5 Kraus W L. New functions for an ancient domain. Nat Struct Mol Biol, 2009, 16: 904–907
6 Karras G I, Kustatscher G, Buhecha H R, et al. The macro domain is an adp-ribose binding module. EMBO J, 2005, 24: 1911–1920
7 Zeldovich K B, Shakhnovich E I. Understanding protein evolution: From protein physics to darwinian selection. Annu Rev Phys Chem, 2008, 59: 105–127
8 Heinen T J, Staubach F, Haming D, et al. Emergence of a new gene from an intergenic region. Curr Biol, 2009, 19: 1527–1531
9 Knowles D G, McLysaght A. Recent *de novo* origin of human protein-coding genes. Genome Res, 2009, 19: 1752–1759
10 Cai J, Zhao R, Jiang H, et al. *De novo* origination of a new protein-coding gene in *Saccharomyces cerevisiae*. Genetics, 2008, 179: 487–496
11 Murzin A G, Brenner S E, Hubbard T, et al. Scop: A structural classification of proteins database for the investigation of sequences and structures. J Mol Biol, 1995, 247: 536–540
12 Kaessmann H. Origins, evolution, and phenotypic impact of new genes. Genome Res, 2010, 20: 1313–1326
13 Moore A D, Bjorklund A K, Ekman D, et al. Arrangements in the modular evolution of proteins. Trends Biochem Sci, 2008, 33: 444–451
14 Babushok D V, Ostertag E M, Kazazian H H Jr. Current topics in genome evolution: Molecular mechanisms of new gene formation. Cell Mol Life Sci, 2007, 64: 542–554
15 Patthy L. Genome evolution and the evolution of exon-shuffling—A review. Gene, 1999, 238: 103–114
16 Enright A J, Iliopoulos I, Kyrpides N C, et al. Protein interaction maps for complete genomes based on gene fusion events. Nature, 1999, 402: 86–90
17 Holm L, Sander C. Parser for protein folding units. Proteins, 1994, 19: 256–268
18 Changolkar L N, Costanzi C, Leu N A, et al. Developmental changes in histone MacroH2A1-mediated gene regulation. Mol Cell Biol, 2007, 27: 2758–2764
19 Buschbeck M, Uribesalgo I, Wibowo I, et al. The histone variant macroH2A is an epigenetic regulator of key developmental genes. Nat Struct Mol Biol, 2009, 16: 1074–1079
20 Araya I, Nardocci G, Morales J, et al. MacroH2A subtypes contribute antagonistically to the transcriptional regulation of the ribosomal cistron during seasonal acclimatization of the carp fish. Epigenetics Chromatin, 2010, 3: 14
21 Chen L, Hu L, Chan T H, et al. Chromodomain helicase/adenosine triphosphatase DNA binding protein 1-like (*CHD1L*) gene suppresses the nucleus-to-mitochondria translocation of NUR77 to sustain hepatocellular carcinoma cell survival. Hepatology, 2009, 50: 122–129
22 Ahel D, Horejsi Z, Wiechens N, et al. Poly(adp-ribose)-dependent regulation of DNA repair by the chromatin remodeling enzyme ALC1. Science, 2009, 325: 1240–1243
23 Wu Z Q, Li Y Z, Li X L, et al. LRP16 integrates into NF-kappaB transcriptional complex and is required for its functional activation. PLoS One, 2011, 6: e18157
24 Friedberg E C. DNA damage and repair. Nature, 2003, 421: 436–440
25 Timinszky G, Till S, Hassa P O, et al. A macrodomain-containing histone rearranges chromatin upon sensing PARP1 activation. Nat Struct Mol Biol, 2009, 16: 923–929
26 Lobell D B, Schlenker W, Costa-Roberts J. Climate trends and global crop production since 1980. Science, 2011, 333: 616–620
27 Umina P A, Weeks A R, Kearney M R, et al. A rapid shift in a classic clinal pattern in *Drosophila* reflecting climate change. Science, 2005, 308: 691–693
28 Ettensohn C A. Lessons from a gene regulatory network: Echinoderm skeletogenesis provides insights into evolution, plasticity and morphogenesis. Development, 2009, 136: 11–21
29 Pinto R, Ivaldi C, Reyes M, et al. Seasonal environmental changes regulate the expression of the histone variant macroH2A in an eurythermal fish. FEBS Lett, 2005, 579: 5553–5558
30 Ladurner A G. Inactivating chromosomes: A macro domain that minimizes transcription. Mol Cell, 2003, 12: 1–3
31 Maynard-Smith J, Harper D. Animal Signals. Oxford: Oxford University Press, 2003
32 Mehrotra P, Riley J P, Patel R, et al. PARP-14 functions as a transcriptional switch for stat6-dependent gene activation. J Biol Chem, 2011, 286: 1767–1776

33 Goenka S, Boothby M. Selective potentiation of stat-dependent gene expression by collaborator of Stat6 (Coast6), a transcriptional cofactor. Proc Natl Acad Sci USA, 2006, 103: 4210–4215
34 Han W D, Zhao Y L, Meng Y G, et al. Estrogenically regulated LRP16 interacts with estrogen receptor alpha and enhances the receptor's transcriptional activity. Endocr Relat Cancer, 2007, 14: 741–753
35 Yang J, Zhao Y L, Wu Z Q, et al. The single-macro domain protein LRP16 is an essential cofactor of androgen receptor. Endocr Relat Cancer, 2009,16: 139–153
36 Han W D, Mu Y M, Lu X C, et al. Up-regulation of LRP16 mrna by 17 beta-estradiol through activation of estrogen receptor alpha (ER alpha), but not ER beta, and promotion of human breast cancer MCF-7 cell proliferation: A preliminary report. Endocr Relat Cancer, 2003, 10: 217–224
37 Roth J, LeRoith D, Shiloach J, et al. The evolutionary origins of hormones, neurotransmitters, and other extracellular chemical messengers: Implications for mammalian biology. N Engl J Med, 1982, 306: 523–527
38 Kimura M. The Neutral Theory of Molecular Evolution. Cambridge: Cambridge University Press, 1983
39 Hasegawa M, Cao Y, Yang Z. Preponderance of slightly deleterious polymorphism in mitochondrial DNA: Nonsynonymous/synonymous rate ratio is much higher within species than between species. Mol Biol Evol, 1998, 15: 1499–1505

**Open Access** This article is distributed under the terms of the Creative Commons Attribution License which permits any use, distribution, and reproduction in any medium, provided the original author(s) and source are credited.
